# Fabrication and Performance of PVAc-Incorporated Porous Self-Standing Zeolite-Based Geopolymer Membranes for Lead (Pb(II)) Removal in Water Treatment

**DOI:** 10.3390/polym17091155

**Published:** 2025-04-24

**Authors:** Samar Amari, Mariam Darestani, Graeme Millar, Bob Boshrouyeh

**Affiliations:** 1 School of Mechanical, Medical & Process Engineering, Faculty of Engineering, Queensland University of Technology (QUT), Brisbane, QLD 4000, Australia; 2 School of Mechanical Engineering, Western Sydney University (WSU), Sydney, NSW 2751, Australia; m.darestani@westernsydney.edu.au; 3 Sustainable Minerals Institute, The University of Queensland, Brisbane, QLD 4072, Australia

**Keywords:** filtration, geopolymer, heavy metal removal, Pb(II) removal, polyvinyl acetate, porous membrane, water treatment, zeolite

## Abstract

This study explores the fabrication, structural characteristics, and performance of an innovative porous geopolymer membrane made from waste natural zeolite powder for Pb(II) removal, with potential applications in wastewater treatment. A hybrid geopolymer membrane incorporating polyvinyl acetate (PVAc) (10, 20, and 30 wt.%) was synthesized and thermally treated at 300 °C to achieve a controlled porous architecture. Characterization techniques, including Fourier-transform infrared spectroscopy (FTIR), revealed the disappearance of characteristic C=O and C-H stretching bands (~1730 cm^−1^ and ~2900 cm^−1^, respectively), confirming the full degradation of PVAc. Thermogravimetric analysis (TG) and differential scanning calorimetry (DSC) indicated a total mass loss of approximately 14.5% for the sample with PVAc 20 wt.%, corresponding to PVAc decomposition and water loss. Energy-dispersive spectroscopy (EDS) elemental mapping showed the absence of carbon residues post-annealing, further validating complete PVAc removal. X-ray diffraction (XRD) provided insight into the crystalline phases of the raw zeolite and geopolymer structure. Once PVAc removal was confirmed, the second phase of characterization assessed the membrane’s mechanical properties and filtration performance. The thermally treated membrane, with a thickness of 2.27 mm, exhibited enhanced mechanical properties, measured with a nano-indenter, showing a hardness of 1.8 GPa and an elastic modulus of 46.7 GPa, indicating improved structural integrity. Scanning electron microscopy (SEM) revealed a well-defined porous network. Filtration performance was evaluated using a laboratory-scale dead-end setup for Pb(II) removal. The optimal PVAc concentration was determined to be 20 wt.%, resulting in a permeation rate of 78.5 L/(m^2^·h) and an 87% rejection rate at an initial Pb(II) concentration of 50 ppm. With increasing Pb(II) concentrations, the flux rates declined across all membranes, while maximum rejection was achieved at 200 ppm. FTIR and EDS analyses confirmed Pb(II) adsorption onto the zeolite-based geopolymer matrix, with elemental mapping showing a uniform Pb(II) distribution across the membrane surface. The next step is to evaluate the membrane’s performance in a multi-cation water treatment environment, assessing the sorption kinetics and its selectivity and efficiency in removing various heavy metal contaminants from complex wastewater systems.

## 1. Introduction

Industrial activities entail a considerable amount of water consumption and, consequently, a substantial amount of wastewater, including a high level of heavy metals. As an example, lead mining produces large amounts of effluent containing Pb(II), which potentially contaminates the environment and freshwater bodies [1]. The detrimental effects of heavy metals on human and animal health make the removal of heavy metals from wastewater and water sources essential. Heavy metals such as Pb(II) are non-biodegradable and accumulate in living organisms, causing toxic effects, including renal dysfunction and neurological disorders, even at low concentrations [1,2]. Due to their severe environmental and health hazards, developing efficient, cost-effective, and sustainable water treatment technologies is of paramount importance [3].

Various techniques have been employed to remove heavy metals from wastewater, including membrane filtration, adsorption, chemical coagulation, solvent extraction, and ion exchange [3]. Membranes, particularly polymeric membranes, dominate wastewater treatment due to their reliability and widespread use. Membrane separation processes, such as electrodialysis, ultrafiltration, nanofiltration, and reverse osmosis, have emerged as effective methods for heavy metal removal from wastewater due to their high efficiency and selectivity [4]. Compared to the conventional methods mentioned above, the membrane process offers advantages, including minimal sludge generation, compact system design, and reduced chemical usage. However, they often face challenges in balancing permeability and selectivity while addressing membrane fouling, high operational costs, scaling, and material instability, particularly in demanding industrial applications. Additionally, fossil-based adsorbents or synthetic membranes can pose environmental or health hazards, necessitating careful selection and disposal strategies [5].

Recently, inorganic membranes have gained significant attention for heavy metal separation [6,7,8,9]. Porous inorganic membranes are highly tunable, typically resistant to organic solvents and high temperatures, chemically stable, cost-effective, reusable, and low-maintenance [9,10]. Additionally, inorganic membranes are less affected by bacterial exposure, which is responsible for the biofouling degradation of most polymeric membranes [9,10]. Based on their material composition and structural properties, inorganic membranes are typically classified into ceramic membranes, including metal oxides, zeolites, and MOFs, as well as carbon-based membranes, such as carbon nanotubes and graphene membranes. [10]. In Table 1, inorganic membranes are compared by their advantages, disadvantages, and applications.

Among these techniques, zeolite membranes, known for their molecular sieving properties, are effective for heavy metal removal from wastewater and gas–liquid separation [10]. Their excellent thermal and chemical stability makes them viable alternatives to organic membranes [11]. Separation occurs through a combination of size exclusion and ion interactions, leveraging the zeolite’s three-dimensional network to selectively adsorb heavy metals [12,13,14,15,16,17,18,19]. These membranes are classified as synthetic or natural; synthetic types, formed on stainless steel or ceramic supports, offer tunable properties but face scalability challenges due to high processing temperatures, intercrystalline voids, and costly supports [11,17,18,19]. Natural zeolite membranes provide a cost-effective alternative but generally exhibit lower water flux [10].

Geopolymer technology, while primarily used in construction [20], has recently gained attention for membrane fabrication due to its low cost, tunable porosity, and high adsorption capacity [21]. Several studies have demonstrated the efficiency of geopolymer membranes in removing toxic heavy metals from aqueous solutions [21]. A ZSM-35 zeolite membrane, synthesized through the geopolymerization of metakaolin and silica fume, achieved 99.22% Cd(II) removal at an initial concentration of 500 ppm under vacuum pressure [22]. A geopolymer–zeolite composite membrane, produced via hydrothermal geopolymerization of fluidized bed fly ash waste, showed 85.45% Cr(VI) rejection [23]. These findings highlight the potential of geopolymer membranes in selective heavy metal adsorption, benefiting from their ion-exchange capability and high surface area [22]. A bio-inspired multilayer geopolymer–chitosan membrane demonstrated 97.77% Cu(II) and 99.4% Pb(II) rejection, alongside the efficient removal of dyes and nanoparticles [23]. The incorporation of chitosan, a natural biopolymer, enhances metal adsorption via chelation mechanisms, making it an effective strategy for improving geopolymer membrane performance [23,24]. Zhang et al. [24] developed a geopolymer–chitosan membrane, achieving 95% removal of triarylmethane dye through a combination of adsorption and molecular sieving. Another geopolymer–chitosan composite was tailored for broad-spectrum contaminant removal, achieving 99.38% crystal violet dye rejection, 99.3% oil-in-water separation, and 98.3% PS nanoparticle removal, suggesting multi-contaminant filtration [25]. Functionalized Cr_2_O_3_-geopolymer membranes have also been explored. A self-supporting Cr_2_O_3_–geopolymer, synthesized from silica fume and metakaolin, was successfully employed for Cr(III) separation before being further processed for dye wastewater treatment [26]. A high-strength, self-supporting NaA zeolite membrane, synthesized through geopolymerization and hydrothermal treatment, achieved 99.5% sodium ion rejection during pervaporation desalination [27]. Another NaA zeolite membrane, fabricated via dip-coating a stainless-steel support with geopolymer paste, was successfully applied for ethanol–water separation under vacuum pressure [28]. A sodalite–geopolymer membrane, produced using kaolinite-rich laterite soils, demonstrated efficient ethanol–water separation, indicating the feasibility of utilizing natural minerals for membrane fabrication [29]. Similarly, analcime–geopolymer composite membranes, supported on sintered sand core slices, exhibited a 95% rejection rate for organic pollutants, such as methylene blue [30]. Beyond water treatment, geopolymer membranes have shown promise in air purification and fuel cell technology. A porous metakaolin-based geopolymer tube membrane achieved 99.5% particulate matter removal, demonstrating its potential in air filtration applications [31]. Additionally, a proton exchange membrane, developed from chitosan–geopolymer composites, has been proposed as a cost-effective alternative to Nafion membranes in fuel cells [32]. The separation mechanism of zeolite-based geopolymer filters is not yet fully understood. However, it is widely recognized as a combination of size exclusion and ion interactions [3]. The geopolymer structure possesses cation exchange capacity, with exchangeable Na(I), K(I), Ca(II), and Mg(II) ions embedded within the framework [33]. When heavy metal cations encounter the filter, they replace these exchangeable cations due to their higher affinity, facilitating the ion exchange process [34]. In a study on the sorption mechanisms and kinetics of both natural zeolite and geopolymer sorbents for Pb(II) and other contaminants using sorption columns, the results revealed that the geopolymer beads exhibited a sorption capacity of 45.32 mg/g, closely matching the 45.93 mg/g recorded for natural zeolite, indicating that the geopolymer retained its adsorption efficiency [3]. Table 2 and Table 3 provide a summary of geopolymer performance in the removal and separation of heavy metals, highlighting their adsorption efficiency, structural properties, and experimental conditions. While cation exchange is the dominant mechanism governing heavy metal sorption, pore architecture also plays a critical role in the separation efficiency [3]. The highly porous structure enhances metal retention by significantly increasing the available surface area and providing micro- and nanoscale pores that facilitate cation entrapment. Within these pores, metal ions diffuse and become physically retained through a combination of van der Waals forces and electrostatic interactions, further enhancing the membrane’s separation performance [35].

Despite advancements, limited studies have explored the use of natural ultrafine zeolite powders (54 microns) to develop flat-shaped, self-standing membranes with controlled porosity and high mechanical strength for heavy metal removal. Most existing commercial applications of zeolites rely on the form and structure of these materials. For example, water treatment using zeolites is usually achieved by passing water across packed bed columns filled with larger particles (0.5–2 mm), a common sorption method [4,5], while the market for ultrafine zeolites remains limited [33]. The geopolymerisation of zeolites can help to solve these problems and promote the performance of zeolites. Compared to other inorganic membrane fabrication methods, this approach appears to be more cost-effective, as it eliminates the need for high-temperature sintering, expensive ceramic supports, and complex multi-step fabrication processes. Additionally, achieving the porosity of inorganic membranes, particularly geopolymer membranes, and controlled pore formation remains a significant challenge. It is vital for membranes to have both high porosity and sufficient mechanical strength, yet these properties are often counterproductive [3,10,33]. Innovative fabrication strategies, such as sacrificial phase removal and tailored geopolymerization processes, offer potential solutions to optimize pore distribution while preserving membrane robustness [36].

**Table 3:** Geopolymer membrane properties and heavy metal removal performances.

Source Material	Contaminant	Strength (MPa)	Curing Time and Temperature	Average Pore Size (nm)	Removal (mg/g)	Water Flux (kg/m^2^h)	Thickness (mm)	Adsorption Conditions	Removal Rate (%)
Metakaolin geopolymer [37]	Ni (II)	18.67	60 °C–24 h	10–1000	22.69–43.36	99.02	10	pH 6 20–23 °C	~95
NaA zeolite geopolymer [27]	Na (I)	57	90 °C–6 h	3.77	--	3.86	<10	--	99.55%
Metakaolin–Silica fume [22]	Cd (II)	--	50 °C–48 h	--	--	26.12	10	--	99.22%
Fly ash–Zeolite [22]	Cr (VI)	12.4	80 °C–24 h	15.99	--	851	6	--	85.45
Silica fume Metakaolin [22]	Cr (III), Dye	--	80 °C–24 h/550 °C–4 h	--	--	22	4	--	100%

The primary objective of this study is to fabricate a porous zeolite-based geopolymer membrane and evaluate its Pb(II) removal efficiency using a laboratory-scale dead-end filtration setup. The hypothesis is that the geopolymerization of natural zeolite can effectively transform ultrafine zeolite powders into a flat-shaped membrane, making them suitable for Pb(II) removal. Second, to achieve controlled porosity, a polymer was incorporated into the geopolymer matrix as a sacrificial phase, representing a novel approach in geopolymer membrane pore formation. This technique involves blending an organic polymer with the inorganic phase, followed by its removal through dissolution or thermal decomposition to create a porous structure. However, for the successful integration into zeolite geopolymers, the polymer must be water-soluble and stable in the highly alkaline geopolymerization environment. In this study, polyvinyl acetate PVAc emulsion at varying concentrations was selected to ensure compatibility with the geopolymer matrix, facilitating tailored porosity while preserving membrane integrity. The major research questions to address include the following: (1) Is it possible to convert zeolite powders into a permeable membrane with sufficient mechanical strength? (2) What are the effects of the initial concentration of Pb(II) ions on water flux and rejection? (3) Would utilizing a water-soluble polymer (i.e., PVAc) create a desirable porous structure? (4) Can the structure and separation properties of membranes be manipulated by PVAc?

## 2. Materials and Methods

### 2.1. Zeolite Geopolymer Membrane Preparation (ZGM)

To prepare the initial geopolymer slurry, 40 g sodium hydroxide solution (40% *w*/*w*, pH: 12.8), acquired from Chem-Supply Pty Ltd. (Gillman, Australia), and 50 g liquid sodium silicate (grade D, SiO_2_/Na_2_O = 1.6–2.6, 55.4–56.4% *w*/*w*, pH: 12.3), acquired from PQ Australia Pty Ltd., Melbourne, Australia, were mixed in a polypropylene beaker. Then, 100 g natural zeolite powder (with an average particle size of 54 μm), supplied from Zeolite Australia Pty Ltd., Sydney, Australia, was added, and the mixture was blended using a mechanical mixer for 15 min. Then, 10 mL PVAc emulsion as the structure-directing agent with different concentrations (10, 20, and 30 wt.%) was added to the slurry (Table 4). The PVAc used in this study was sourced from Sigma Aldrich, Melbourne, Australia, with an average molecular weight of 100,000 g/mol and a density of 1.18 g/cm^3^, provided in bead form. The PVAc emulsion was selected due to its water solubility and its non-altering effect on the pH of materials it contacts. A 30 wt.% PVAc emulsion was prepared by dissolving 30 g of PVAc beads in 40 mL of ethyl acetate under continuous stirring at room temperature using a magnetic stirrer at 500 rpm, followed by gentle heating to 50 °C until fully dissolved. During preparation, the relative humidity was 40–60%. The resulting solution was then slowly added to 70 g of deionized (DI) water while stirring at a high speed of 1000 rpm to promote emulsification. Finally, the mixture was heated to 50–60 °C under vacuum to evaporate the solvent, yielding a stable PVAc emulsion with 30% wt. To obtain 20 wt.% and 10 wt.% emulsions, the 30 wt.% emulsion was diluted with DI water in appropriate proportions while stirring continuously until homogeneous.

**Figure 1:**
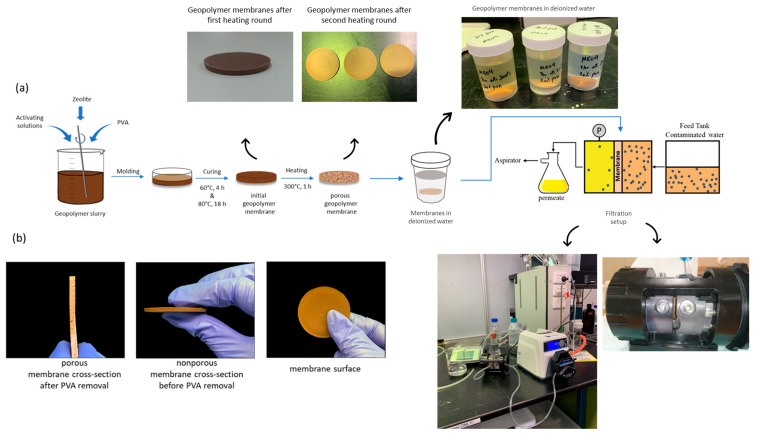
(**a**) Schematic of ZGM membrane production process and the filtration setup. (**b**) Surface and cross-section of non-porous and porous cross-section of ZGMs.

Next, 8 g of the well-mixed slurry were cast into polypropylene Petri dishes with a diameter of 50 mm. The ZGMs were cured first at 60 °C for 4 h and then at 80 °C for 18 h and demolded after cooling. To create a porous structure, PVAc must be removed from the geopolymer matrix, following an approach inspired by the phase inversion technique used in organic polymer membranes [37]. In this process, the hypothesis is that the thermal decomposition of PVAc at high temperatures facilitates the formation of interconnected pores, enhancing the porosity and surface area of the geopolymer membrane. To remove PVAc from the ZGMs and create a porous matrix, the samples were annealed at a temperature of 300 °C for 1 h. This temperature was chosen because a TGA analysis confirmed that PVAc decomposed at around 300 °C. The prepared membranes were immersed in deionized water before being applied for filtration tests. The ZGMs membrane production process and the filtration setup are shown in Figure 1.

### 2.2. Water Flux Experiments

**Figure 2:**
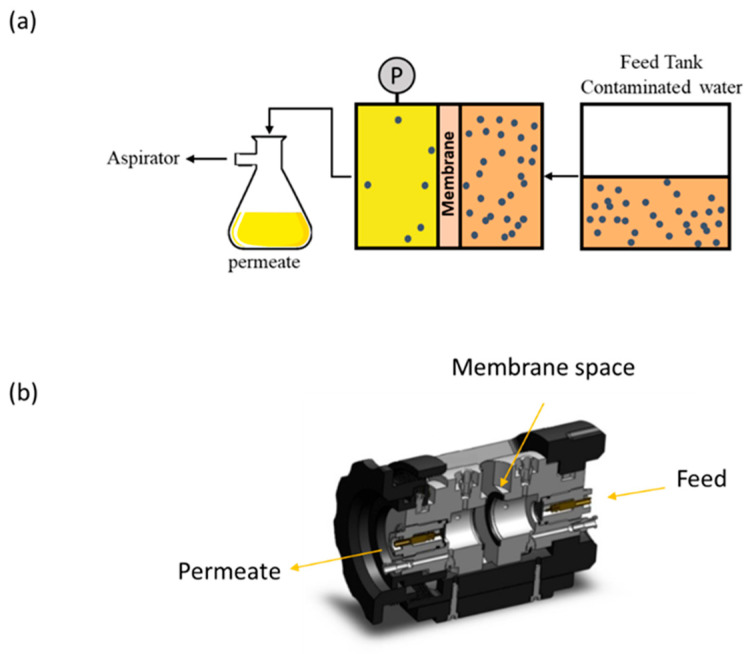
(**a**) Schematic illustration of the filtration process and (**b**) cross-sectional view of the two-chamber membrane setup operated at room temperature and pH 6.

To evaluate water flux and contaminant removal performance, the ZGM membranes were mounted into a custom-designed membrane filtration setup (Figure 2b), which comprised two separate compartments. A constant vacuum pressure of 0.1 MPa was applied throughout the filtration experiment. The filtrate was collected at designated time intervals. Water flux (J) through the ZGMs was determined using the following equation:

J = m/(A × Δt)(1)

where J represents the water flux (kg·m⁻^2^·h^−1^), m is the mass of water permeated through the membrane, A is the effective surface area of the membrane, and Δt is the duration of the filtration process. A schematic representation of the filtration system is shown in Figure 2a [38].

Different concentrations of Pb(II) solutions (50, 100, and 200 ppm) were prepared using lead(II) chloride (PbCl_2_), supplied by Sigma Aldrich (USA). Accurately weighed amounts of PbCl_2_ were dissolved in deionized water under continuous stirring to ensure complete dissolution at room temperature. A stock solution of 1000 ppm Pb(II) was first prepared by dissolving a known mass of PbCl_2_ in deionized water. The final volumes were adjusted to the desired concentrations in volumetric flasks. The pH of each Pb(II) solution (50 ppm, 100 ppm, and 200 ppm) was monitored using a calibrated pH meter. The initial pH measurements of the Pb(II) solutions were 5.3 for 50 ppm and 4.08 for 200 ppm. Small volumes of 0.1 M NaOH were gradually added dropwise under constant stirring until the desired pH value of 6.0 ± 0.2 was reached. After pH adjustment, the solutions were equilibrated for 10 min to ensure pH stability before being used in membrane filtration experiments. pH was controlled at neutral conditions (pH~6) to avoid additional complexities, such as metal precipitation or membrane degradation, which may occur under extreme pH conditions. Temperature was maintained at room temperature (25 ± 2 °C) to mimic realistic environmental conditions and eliminate the temperature effect on the diffusion rates. The concentration of Pb(II) in the feed and permeate solutions was analyzed using an inductively coupled plasma–optical emission spectroscopy (ICP-OES) analyzer, and the removal efficiency was calculated by the next equation:

(2) $$R=\frac{C_{0}-C_{1}}{C_{0}}\times 100\%$$

where C_0_ and C_1_ are the heavy metals’ concentrations in the feed and permeate solutions, respectively [38].

### 2.3. Characterization Techniques

#### 2.3.1. X-Ray Diffraction

X-ray diffraction analysis was performed to characterize the crystalline phases present in the zeolite and geopolymer samples. A PANalytical Pro diffractometer (Almelo, The Netherlands), operating at 40 kV and 40 mA with a cobalt (Co) radiation source, was used for the measurements for sample preparation. An accurately weighed portion of the geopolymer sample was mixed with a specific weight percentage of corundum (Al_2_O_3_, Baikowski International), which served as an internal standard. To ensure homogeneity, 10 mL of ethanol were added to the mixture, which was then micronized using a McCrone mill with zirconia beads for six minutes. The resulting slurry was dried in an oven at 40 °C for 12 h. Once dried, the uniform powders were backloaded and pressed into sample holder disks for analysis. During the XRD measurement, the samples were rotated to improve the counting statistics. The instrument was configured with the following incident optics: a 15 mm mask, 0.04 radian Soller slits, a 0.5° fixed divergence slit, and a 2° fixed anti-scatter slit. Data were collected over a 2θ range starting at 5°, using a step size of 0.016°, with a total measurement time of 30 min per sample. Phase identification was conducted using the PANalytical HighScore Plus (Version 4) and MDI Jade (Version 4.1) software packages, with reference to multiple crystallographic databases, including PDF4+, the American Mineralogist Crystal Structure Database (2010), the Crystallographic Open Database, and ICSD FIZ Karlsruhe (2011). Quantitative phase analysis was performed using the Rietveld refinement method, as implemented in TOPAS (Version 5, Bruker, Billerica, MA, USA) [33].

#### 2.3.2. Fourier Transform Infrared Spectroscopy

Fourier-transform infrared (FTIR) spectra were obtained using a Nicolet iS50 spectrometer (Thermo Scientific, Madison, WI, USA) equipped with a single-bounce diamond ATR accessory. Each spectrum was collected by averaging 64 scans at a resolution of 4 cm^−1^ [39].

#### 2.3.3. Thermogravimetric/Differential Scanning Calorimetry

Thermal analysis, including differential scanning calorimetry (DSC) and thermogravimetric analysis (TGA), was carried out using a STA 449F3 TG-DSC system (Netzsch, Germany). The measurements were performed under a nitrogen atmosphere, applying a heating rate of 10 °C/min to examine the thermal characteristics of the raw materials and synthesized geopolymers [33].

#### 2.3.4. Inductively Coupled Plasma–Optical Emission Spectroscopy

Multi-element quantitation was determined using a Perkin Elmer 8300DV (Shelton, Connecticut, USA), ICP-OES, which was coupled with an ESI SC-4DX autosampler and a PrepFAST 2 sample introduction system for automated dilution and online internal standardization. All standards and blanks were prepared using purified nitric acid. Calibration involved in-house prepared multi-element standards derived from ICP-MS-grade single-element stock solutions (High Purity Standards, Charleston, CA, USA). The elements typically calibrated included aluminum, boron, calcium, cadmium, cobalt, chromium, copper, iron, potassium, lithium, magnesium, manganese, sodium, sulfur, silicon, strontium, and zinc—commonly found in environmental water samples. Method accuracy and reproducibility were confirmed through the regular analysis of certified reference materials (CRMs) from NIST (Gaithersburg, MD, USA) and the US Geological Survey (Reston, VA, USA). Additionally, certified wastewater samples trace metals A, B, and D (CWW-TM-A, CWW-TM-B, and CWW-TM-D) were used for non-routine validation, while initial calibration verification standards (ICV-1A and ICV-1B) were used routinely across a wide concentration range [40].

#### 2.3.5. Surface Area and Pore Size Distribution

The specific surface area and pore distribution of the samples were analyzed using BET (Brunauer–Emmett–Teller) and BJH (Barrett–Joyner–Halenda) methods via a Micromeritics 3Flex Version 4.05 analyzer. (Micromeritics, Norcoss, GA, USA) Prior to measurement, the samples were degassed at 250 °C under vacuum for 48 h. Nitrogen adsorption isotherms were recorded, and the resulting data were processed both manually and using the MicroActive software suite (version 7) [41].

#### 2.3.6. Hardness and Elastic Modulus Analyses

The nano-mechanical properties, including hardness and elastic modulus, were measured using a Hysitron TI 950 TriboIndenter (Bruker, Billerica, MA, USA). The Oliver–Pharr method was employed to determine the hardness (H) and reduced elastic modulus (Er) from the loading curve up to the maximum indentation depth [42].

#### 2.3.7. Scanning Electron Microscopy/Energy-Dispersive Spectroscopy

Sample morphology and surface elemental composition were analyzed using a JEOL 7001F scanning electron microscope (Tokyo, Japan), coupled with energy dispersive spectroscopy (EDS). Samples were mounted on conductive carbon adhesive tabs and sputter-coated with 99.99% pure gold using a Leica EM SCD005 sputter coater under argon gas, at a chamber pressure of 0.05 mbar and a current of 30 mA. SEM observations were conducted at an accelerating voltage of 10 kV, and secondary electron images were captured at multiple magnifications [33].

## 3. Results and Discussion

### 3.1. Zeolite and ZGMs Characterization

**Figure 3:**
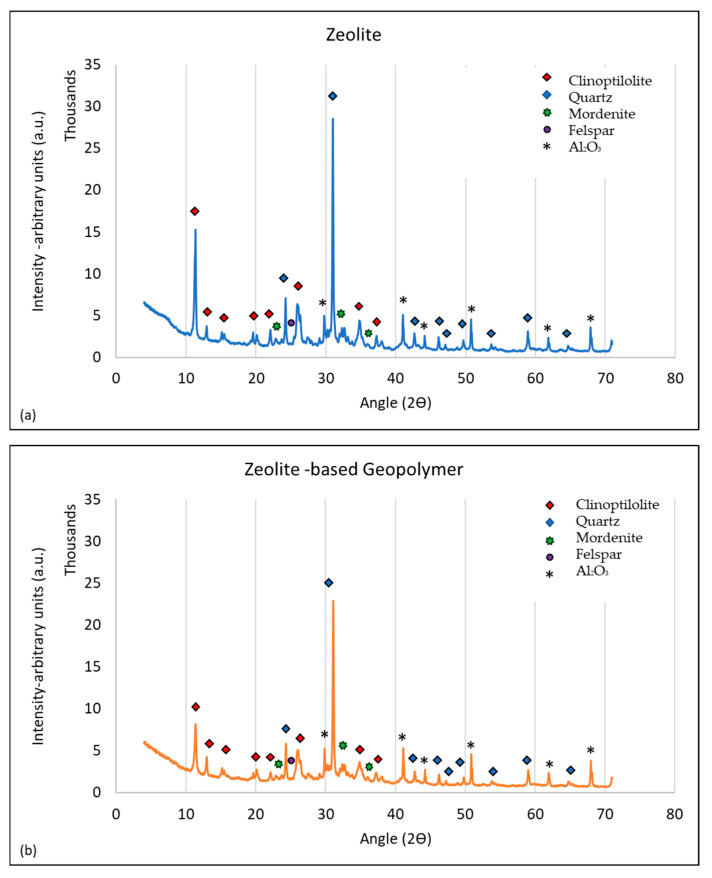
XRD patterns of (**a**) zeolite and (**b**) zeolite-based geopolymer. The diffraction peaks are labeled using color-coded shapes to indicate the identified crystalline phases: clinoptilolite (red diamond), quartz (blue diamond), mordenite (green hexagon), feldspar (purple circle), and Al_2_O_3_ (black asterisk).

The XRD patterns of both ZGM and natural zeolite are presented in Figure 3. The quantitative analyses, together with the crystal powder diffraction number (PDF#), can be found in Table 5. In brief, the natural zeolite with a cation exchange capacity of 134.6 meq/100 g [42] consisted of clinoptilolite–heulandite (~28.1%), with an International Zeolite Association framework code of HEU, quartz (~20%), stilbite (~19.1%) with framework type of STI, and minor amounts of feldspar, mordenite, plagioclase and chabazite. About 21.5% of the zeolite was unidentified, including amorphous components such as amorphous SiO_2_ [43] and a trace amount of smectite. Quantitative XRD analysis revealed a significant reduction in quartz content (up to ~50%) during geopolymer development, as well as a decrease in the clinoptilolite–heulandite content (up to ~75%). Approximately 95% of stilbite was removed from the geopolymer matrix. Albite, andesine, feldspar, and mordenite also diminished (by approximately 60%), while a new phase, anorthite (plagioclase), was formed during the geopolymerization reaction. Overall, the amorphous content increased from ~21% to ~73%, which was expected due to the geopolymerization process and is consistent with findings by Ulloa et al. [44]. Amari et al. [33] also observed an increase in amorphous content in zeolite-based geopolymers, which they attributed to the disruption of the original crystalline framework during alkali activation.

During the initial slurry preparation, PVAc is introduced in the form of an emulsion and is evenly dispersed throughout the geopolymer matrix. Its interaction with the geopolymer components occurs at multiple levels. First, physical dispersion in the geopolymer slurry occurs. Since PVAc is water-soluble in its emulsified form, it is homogeneously distributed in the geopolymer mixture. PVAc has minimal chemical interaction with the geopolymer network, and its presence does not alter the pH or disrupt geopolymerization, allowing the gelation and crosslinking of the aluminosilicate framework to proceed as normal. As the geopolymer matrix forms, PVAc molecules become physically trapped within the network, forming polymer-rich domains within the rigid inorganic framework.

**Figure 4:**
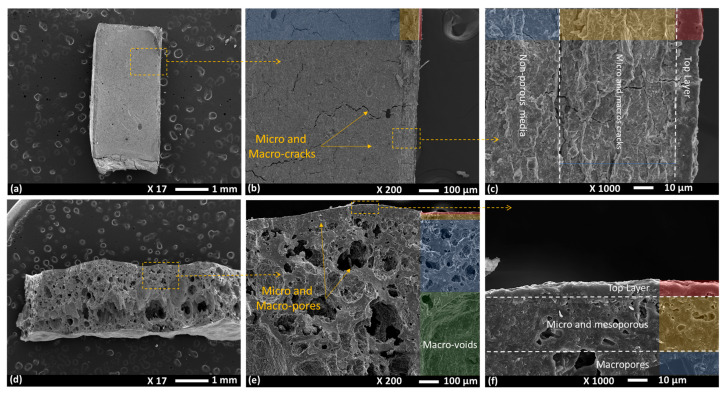
Cross-sectional SEM images of zeolite-based geopolymer membranes at different magnifications: (**a**–**c**) structure before PVAc decomposition, highlighting the presence of micro- and macrocracks; (**d**–**f**) membranes after PVAc decomposition at 300 °C, demonstrating enhanced porosity with distinct micro-, meso-, and macroporous regions.

ZGMs, before PVAc removal, exhibited low porosity. The SEM image (Figure 4) of the geopolymer samples before PVAc decomposition shows the membrane being impermeable, with micro- and macrocracks within the structure, resulting in a weak and fragile membrane. The cracks originated from the top layer, which had a depth of ~10 µm, and extended through the intermediate (~58 µm) and non-porous layers, with the most severe cracking observed in the intermediate zone. The top (dense) layer, which was directly exposed to air, formed due to rapid phase inversion. During the first drying cycles at 60 °C and 80 °C for 22 h, since the organic additive (i.e., PVAc) has a significantly higher decomposition temperature, only water is removed during the drying stage. In this initial phase, interstitial water occupying the spaces between particles is eliminated, resulting in the first stage of membrane shrinkage and microcrack formation, as can be seen in Figure 4b. During the first round of drying, 2.25–2.32 g of water was removed, leading to a linear shrinkage of 28.1–29%, calculated using the mass loss across samples with varying PVAc contents. The 10 wt.% PVAc sample exhibited the highest shrinkage (~29%), while the 30 wt.% PVAc sample showed the least (~28.13%). The 20 wt.% PVAc exhibited moderate shrinkage of 28.63%. This trend suggests that higher PVAc concentrations contribute to greater structural stability, reducing the relative proportion of water available for evaporation-induced shrinkage and thereby minimizing overall contraction.

Once the geopolymer membranes are cured, the removal of PVAc via thermal decomposition (annealing at 300 °C) is critical to generating porosity. The phase-inversion mechanism relies on PVAc acting as a sacrificial phase that, upon decomposition, leaves behind interconnected voids. At this stage, the interaction is predominantly physical rather than chemical. Initially dispersed polymer domains become sites of structural evolution as they shrink, vaporize, and escape the geopolymer matrix. Because PVAc has minimal chemical interaction and negligible reactivity with the geopolymer network, the inorganic aluminosilicate structure remains stable and unaffected chemically. However, physically, as PVAc decomposes and exits, the geopolymer structure experiences localized shrinkage stress and the formation of internal pores. The process unfolds in several steps, starting with softening and thermogravimetric degradation at around 250–300 °C, and breaking down into smaller volatile compounds (e.g., acetic acid, acetone, carbon dioxide, and water vapor). The removal of PVAc results in the formation of pores, where the polymer-rich regions previously existed with different levels of extensivity depending on the initial concentration of PVAc in the formulation. During the second round of heating at 300 °C, additional mass loss occurred, primarily due to PVAc decomposition and the removal of residual moisture, as confirmed by the TG-DSC analysis. The 30 wt.% PVAc sample exhibited the greatest mass loss (2.80 g) and shrinkage (48.70%), corresponding to its higher polymer content. This extensive degradation of PVAc facilitated the formation of a more interconnected and porous network, resulting in larger pore volumes, as evidenced by the SEM micrographs (Figure 4). The 20 wt.% PVAc sample showed a moderate mass loss of 1.95 g, with a corresponding shrinkage rate of 34.15%. This indicates the development of a well-distributed pore structure with moderate porosity, striking a balance between pore formation and structural integrity. In contrast, the 10 wt.% PVAc sample demonstrated the lowest mass loss (1.10 g) and shrinkage (19.37%), reflecting its lower polymer content. This resulted in a more compact structure with fewer and smaller pores, preserving a higher degree of structural stability following thermal treatment.

The molecular weight of the organic additive significantly influences the characteristics of the resulting porous geopolymer. In general, a high molecular weight forms longer polymer chains, leading to more entangled networks within the geopolymer matrix and increasing the viscosity, while a lower molecular weight would disperse more uniformly and may create smaller, less-interconnected pores. The polymer chain structure can also affect pore morphology, influencing the permeability and surface area of the membrane. Linear chains tend to form elongated pores when removed, while branched structures may result in less uniform, more tortuous pore networks. However, the polymer chain environment significantly influences its structure and performance within the geopolymer matrix, with pH and temperature being key factors. For example, in a highly alkaline geopolymer slurry (pH~12.8), polymer chains with acidic functional groups readily ionize, leading to strong electrostatic repulsion between neighboring carboxylate groups [45]. This causes the chains to extend and adopt a more linear conformation. In this study, PVAc was selected because it lacks acidic functional groups and remains neutral under these conditions. In this study, the polymer molecular weight and chain structure were held constant. Therefore, the influence of varying these parameters on the pore morphology and membrane properties was not experimentally explored. Future studies may focus on systematically varying the polymer molecular weight and branching to fully elucidate their effects on pore formation and membrane performance.

**Figure 5:**
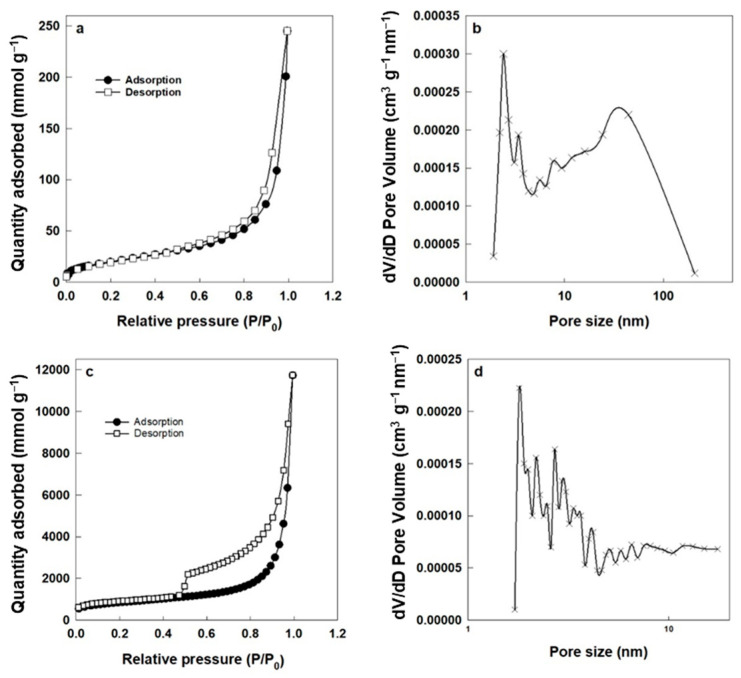
N_2_ gas adsorption–desorption isotherms of ZGM3 membranes (**a**) before and (**c**) after heating at 300 °C, and corresponding pore size distribution histograms (**b**) before and (**d**) after heating at 300 °C, highlighting changes in porosity and pore structure due to PVAc removal.

The Brunauer–Emmett–Teller (BET) isotherm method was employed to calculate the surface area of the membranes before and after thermal annealing. Based on IUPAC classification, the adsorption–desorption isotherm corresponds to Type IV [46]. The hysteresis loop occurred in the range of ~0.6 to 1.0 P/P_0_, indicating the presence of mesopores (Figure 5a,c). Notably, the hysteresis loop observed was closed, suggesting that the adsorption and desorption processes were largely reversible and that the pore structure was stable, with minimal pore blocking or cavitation effects. The closed loop further implies that the pores have uniform connectivity and that there are no significant ink-bottle-shaped pores, which can lead to delayed evaporation and typically result in an open loop. This reversibility is advantageous for consistent performance in applications such as separation membranes, as it reflects a robust pore architecture [47]. The N_2_ gas adsorption–desorption isotherm loop before and after heating is shown in Figure 5b. The specific surface area of the membrane before heat exposure was 4.26 m^2^/g, which increased significantly to 118.3 m^2^/g for the ZGM membrane after PVAc removal. This surface area exceeds the values reported by He et al. [27], who achieved 97.8 m^2^/g in geopolymer–zeolite composite membranes. Our higher surface area may be attributed to the effective sacrificial role of PVAc, which, upon decomposition at 300 °C, created a more interconnected mesoporous network. Similar surface areas (24.59 m^2^/g) were reported by Song et al. [25] in biomimetic multilayer geopolymer membranes. However, their surface area was influenced by multiple factors, including spin-coating conditions, the composition and concentration of the geopolymer precursors, and the number of deposited layers. Additionally, the drying process and post-treatment steps, such as alkali activation and curing temperature, played significant roles in determining the final porosity and specific surface area of the membranes. In our study, PVAc initially occupied the pore spaces within the membrane structure, limiting the accessible surface area. Upon heating, PVAc was thermally degraded and eliminated, leaving behind additional voids and opening previously blocked pore channels. This process not only created new pores but also enhanced the interconnectivity of the existing pores, resulting in a significant increase in both micropore and mesopore volume. Specifically, the percentage of micropores and mesopores in ZGM3 increased slightly (to 10% and 53%, respectively), while the macropore percentage decreased to 37%. This shift from macropores to more micropores and mesopores contributed to the substantial increase in the specific surface area, as smaller pores offer more surface area per unit volume.

According to the Barrett–Joyner–Halenda (BJH) pore size distribution curve of the sample before heating (Figure 5b), about 6% of the total pore volume belonged to micropores (<2 nm), represented by a small narrow peak at ~2 nm. Additionally, 50% and 44% of the total volume were attributed to mesopores (2–5 nm) and macropores (>5 nm), respectively [40,46]. The peaks at 2–3 nm and 5–7 nm can be associated with vacant spaces between crystals and the geopolymer matrix. The broad peak in the range of 10–21 nm and the strong peak at 32 nm may be due to void formation during geopolymerization [41]. After heating, the redistribution of pore sizes, with enhanced microporosity and mesoporosity and reduced microporosity, contributed to a higher total surface area. These results indicate that removing PVAc as a structure-directing agent enhanced the micropore and mesopore content, significantly reducing the number of macropores in ZGM3 and leading to a substantial increase in specific surface area. In gas and liquid separations, both micropores and mesopores play a critical role [40].

The porosity of the membranes was significantly enhanced by the thermal decomposition of the organic component, PVAc, at 300 °C [48]. In conventional inorganic membrane fabrication, a porous substrate is generally required, which can be expensive, and a calcination step is necessary to ensure bonding between the support and the selective layer. In contrast, porous ZGM membranes possess a hierarchical graded structure (Figure 4d), consisting of layers with varying pore sizes, namely, a macroporous layer with pore sizes exceeding 5 µm, a microfiltration layer ranging from 0.1 to 5 µm, and an ultrafiltration layer with pore sizes below 0.1 µm [48]. The topmost layer is the densest, and pore sizes increase progressively toward the inner layers. This gradation effectively reduces the accumulation of permeates in the preceding layers, thereby minimizing the risk of pore blockage [48].

**Figure 6:**
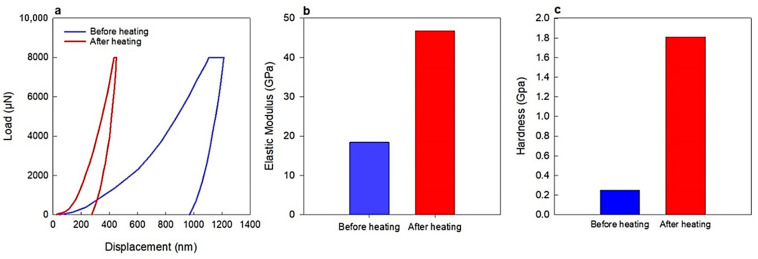
Mechanical properties of membranes before and after heating at 300 °C: (**a**) Load–displacement curves, (**b**) elastic modulus, and (**c**) hardness, demonstrating the improvement in structural properties after thermal treatment.

An important advantage of the developed membranes was their enhanced mechanical strength. Although it was expected that ZGMs might exhibit reduced strength due to increased porosity and the effects of thermal stress [48], the hardness of the material significantly improved following annealing. Remarkably, thermal treatment also led to the healing of micro- and macrocracks previously observed in SEM analyses. The fracture behavior of the membranes, both before and after thermal annealing, was evaluated using a nano-indentation system. Hardness and elastic modulus values were derived from the load–displacement curves (Figure 6). Before heating, the hardness and elastic modulus were measured at 0.25 and 18.42 GPa, respectively. These values increased significantly after annealing, reaching 1.81 and 46.75 GPa. The obtained mechanical strength surpassed that reported in earlier research. For example, Chen et al. [49] synthesized a metakaolin-based geopolymer and reported maximum hardness and modulus values of 0.14 and 3.16 GPa, respectively. The comparatively lower mechanical properties in that study were mainly attributed to a low curing temperature (60–80 °C) and a short curing duration (14 days), which restricted the extent of geopolymerization, yielding a structure with lower density. It is well-established that higher curing temperatures and prolonged curing times enhance mechanical strength by promoting more complete gel formation and reducing overall porosity. Additional factors, such as elevated water content and variation in the Si/Al ratio, also influence mechanical performance by contributing to a less compact structure [49]. As evident from the indentation data, the unheated samples exhibited greater penetration depth, reflecting a softer material. The improved hardness after annealing is likely due to continued geopolymerization at elevated temperatures, promoting particle fusion and bonding within the inorganic phase, which contributes to increased mechanical rigidity [50].

Another notable advantage of these membranes was their high mechanical strength. Although it was anticipated that ZGMs would experience a reduction in strength due to increased porosity and thermal stresses [48], the hardness improved considerably. More significantly, the micro- and macrocracks detected by SEM were healed after the thermal annealing. The fractural behaviors of ZGMs before and after annealing were measured using a nano-indenter instrument. The hardness and elastic modulus of the membranes were determined using the load–distance curve (Figure 6). The initial hardness and elastic modulus were 0.25 and 18.42 GPa before heating and increased drastically to 1.81 and 46.75 GPa, respectively, when the membranes were annealed. This mechanical strength exceeds the values reported in previous studies. For instance, Chen et al. [49] synthesized a metakaolin-based geopolymer and found the maximum hardness and elastic modulus to be 0.14 and 3.16 GPa, respectively. The lower hardness and Young’s modulus values in this study are primarily due to the combined effect of low curing temperature (60–80 °C) and short curing time (14 days), which limited geopolymerization and resulted in a less dense structure. Higher temperatures and longer curing are known to improve mechanical strength by enhancing gel formation and reducing porosity. This is further influenced by factors such as higher water content and variations in the Si/Al ratio, which also contribute to reduced mechanical strength [49]. As can be seen, the indentation depth was higher before thermal treatment, indicating a softer structure. Increased hardness after annealing can be partly attributed to the continuation of geopolymer reaction at higher temperatures, which can be due to the fusion and bonding of the particles occurring in the inorganic phase, which improves the mechanical hardness [50].

**Figure 7:**
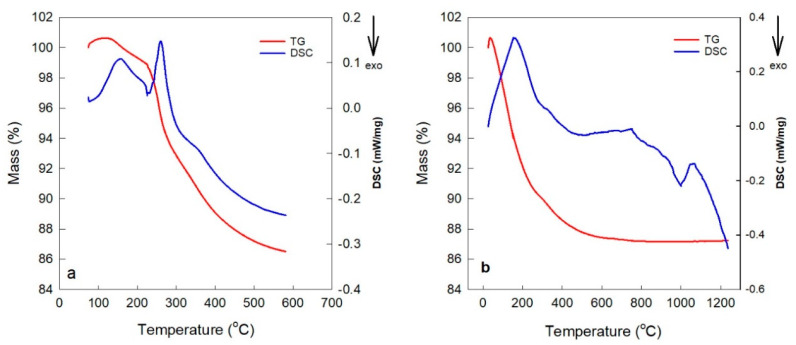
TG-DSC analysis curves for membranes containing PVAc: (**a**) first heating cycle from 50 °C to 600 °C at a rate of 10 °C/min under air atmosphere, and (**b**) second heating cycle from room temperature to 1200 °C at a rate of 10 °C/min.

It was hypothesized that residual PVAc particles could contribute to enhanced mechanical strength. To verify complete polymer removal following thermal treatment, a range of analytical techniques was utilized. Initially, thermogravimetric and differential scanning calorimetry (TG-DSC) analyses were conducted on membranes containing PVAc, gradually heating the samples from 50 °C to 600 °C at a rate of 10 °C/min in an air atmosphere to observe mass loss and thermal transitions (Figure 7a). After cooling the sample at room temperature for 24 h to allow for moisture adsorption, a second heating cycle was applied from ambient temperature to 1200 °C at the same rate (Figure 7b). A slight increase in mass in the TG curves during the early stages of heating—prior to significant weight loss—was detected in both curves (Figure 7a,b), likely due to oxidation of residual organics or reduced metal species, or surface adsorption of atmospheric moisture or oxygen. These behaviors are commonly observed in TG studies under oxidative conditions. The total mass loss recorded was 14.5% in the first cycle and 12% in the second. The initial mass loss was primarily attributed to the release of absorbed water and thermal decomposition of PVAc within the geopolymer framework, while the latter was mostly due to water evaporation. A distinct change in the slope of the TG curve between ~250 °C and ~300 °C during the first cycle, accompanied by an endothermic peak in the DSC curve, corresponded to PVAc decomposition. This behavior was absent in the second cycle, confirming that PVAc degradation had occurred in the first. PVAc decomposes in this temperature range through deacetylation, releasing volatile products such as acetic acid, followed by polymer backbone breakdown. Additional water loss and the degradation of residual organics also contribute to this mass change. These findings support that PVAc was fully eliminated from the geopolymer matrix below 300 °C. A minor DSC peak at ~300 °C in the second cycle is attributed to the evaporation of mesoporous water, which was likely masked by the decomposition peak in the first cycle.

**Figure 8:**
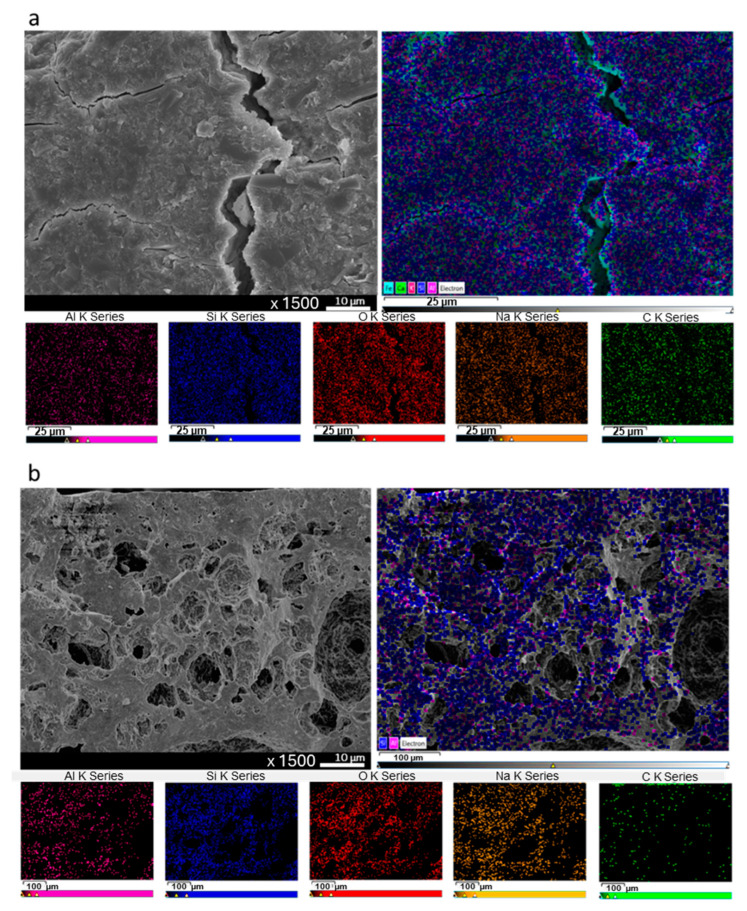
SEM images and corresponding EDS elemental mapping of membrane cross-sections: (**a**) before heating at 300 °C and (**b**) after heating, showing changes in morphology and elemental distribution due to thermal treatment.

To further confirm PVAc removal, EDS elemental mapping of ZGM membranes containing PVAc was performed before and after thermal treatment (Figure 8). The mapping consistently detected Si, Al, Ca, O, and Na across the scanned areas in both cases. Carbon mapping, indicated by green signals, showed a dense and widespread distribution of carbon in the unheated sample (Figure 8a), while only sparse carbon signals were present after heating (Figure 8b), indicating significant polymer elimination. Since PVAc was the sole carbon-containing component in the matrix, these EDS results confirmed its substantial decomposition. The residual carbon observed could be due to remaining ash or trace amounts of undecomposed carbon species, rather than intact PVAc. Enhancing the annealing duration or increasing the thermal treatment temperature could assist in eliminating these traces completely.

**Figure 9:**
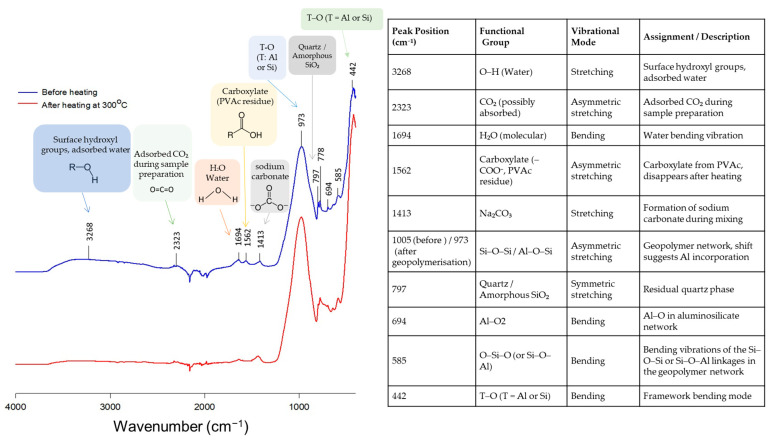
FTIR spectra of ZGM membranes before heating (blue line) and after heating at 300 °C (red line). The annotated peaks indicate the functional groups identified at specific wavenumbers and their corresponding vibrational modes.

FTIR spectroscopy was employed to assess the structural changes in the geopolymer matrix before and after heating at 300 °C (Figure 9). The IR spectra revealed characteristic peaks found in natural zeolite and geopolymer materials. The peak at 442 cm^−1^ corresponds to T–O (T = Al or Si) bending vibrations [33,43], while the signal at 694 cm^−1^ is associated with AlO_2_ functional groups [51]. A band near 797 cm^−1^ indicates the presence of quartz or amorphous SiO_2_ within the zeolite [43], and these fingerprint peaks were also identifiable in the XRD profiles of the samples, supporting the conclusion that the crystal structure remains largely intact through geopolymerization, despite compositional changes. The broad band around 3268 cm^−1^ and the peak at 1694 cm^−1^ are attributed to the stretching and bending vibrations of water molecules, respectively. Water exhibits O–H stretching in the 3200–3600 cm^−1^ range and H–O–H bending around 1600–1650 cm^−1^. A sharp peak, originally observed at 1005 cm^−1^ in natural zeolite, shifted to 973 cm^−1^ after geopolymer formation. This band corresponds to Si–O–Si or Al–O–Si stretching vibrations [52,53]. The observed shift to a lower wavenumber indicates an increase in bond length or a reduction in bond angle, which is commonly used as an indirect measure of aluminum incorporation into the silicate network [53].

A peak at 1413 cm^−1^ corresponds to the formation of sodium carbonate, which occurs due to the reaction between sodium hydroxide and atmospheric carbon dioxide during the mixing of the geopolymer slurry. In the FTIR spectra of the membranes, a prominent peak at 1562 cm^−1^ was evident prior to thermal treatment and is attributed to the carboxylic acid functional groups present in PVAc [54]. This peak completely disappeared after heating, indicating the decomposition and elimination of PVAc from the geopolymer structure. Although the removal of PVAc affected the membrane’s porosity characteristics, it had no notable influence on the fundamental chemical composition of the geopolymer membrane.

### 3.2. Membrane Performance

The performance of zeolite-based geopolymer membranes is based on a combination of size exclusion and ion interactions, both of which are influenced by membrane porosity. While cation exchange plays a well-established role in heavy metal removal, the relationship between membrane porosity and separation performance remains less understood. To enhance porosity and surface area, which are crucial for both adsorption and filtration efficiency, PVAc was introduced as a sacrificial phase in the geopolymer matrix. To systematically investigate these effects, ZGMs were synthesized with varying porosity levels by adjusting the PVAc concentrations, allowing for controlled pore formation. Their Pb(II) separation efficiencies were evaluated under different operating conditions to determine the influence of porosity on flux rate, rejection efficiency, and adsorption performance. Additionally, the effect of the initial Pb(II) concentration on membrane performance was analyzed to understand its impact on membrane permeability.

#### 3.2.1. Influence of PVAc Concentration on Membrane Porosity and Filtration Performance

By varying the initial PVAc concentration from 10 wt.% to 30 wt.%, significant changes were observed in the porosity and microstructure of ZGMs, directly impacting their flux and rejection performance. The separation performance of the membranes without polymer addition could not be assessed due to insufficient mechanical strength, making them unable to withstand the applied pressure in the permeation test.

**Figure 10:**
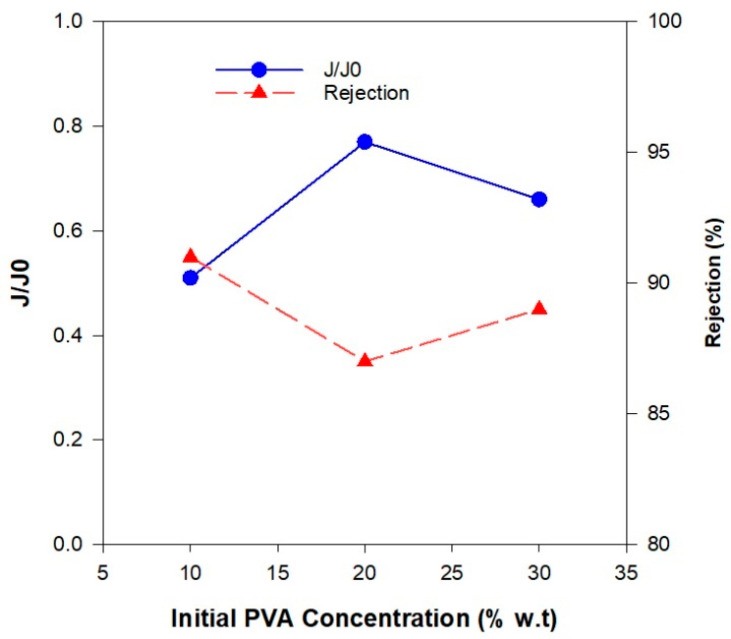
Normalized membrane flux (J/J0, blue circle) and rejection performance (red triangles) of ZGM, evaluated at a constant initial Pb(II) ion concentration of 50 ppm, room temperature, and neutral pH.

As shown in Figure 10, the normalized flux value (i.e., the ratio of permeate flux to initial water flux) was the lowest for the membrane with 10 wt.% PVAc, which corresponds to its lower porosity. Despite exhibiting the lowest flux, this membrane achieved the highest Pb(II) rejection rate of 91% when tested with a 50 ppm Pb(II) solution. However, high rejection efficiency alone is not sufficient for practical applications; an industrially viable membrane must also maintain an adequate flux to ensure efficient water permeability. The poor permeability of this membrane increased the contact time between the water and the membrane surface, allowing for more effective contaminant separation and adsorption.

**Figure 11:**
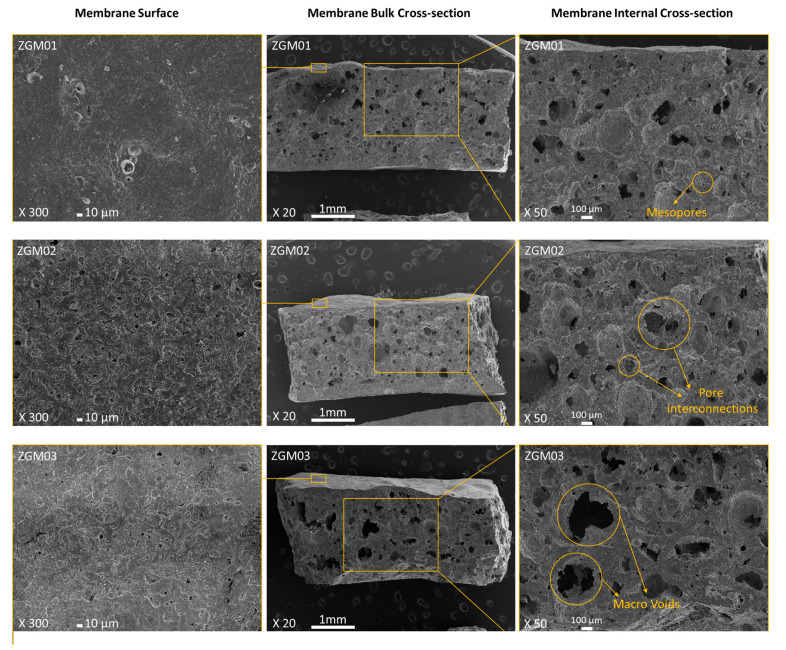
SEM micrographs of zeolite geopolymer membranes prepared with different initial PVAc concentrations (%), shown at various magnifications: membrane surface morphology, bulk cross-section, and internal cross-section.

ZGM2 (10 wt.% PVAc) exhibited a low degree of pore interconnectivity, with only a few observable pores on the surface. Cross-sectional micrographs confirmed a low void content and poor interconnection among the pores. This restricted water permeability, resulting in low flux but high Pb(II) rejection efficiency. The reduced flux was attributed to the limited transport pathways for water molecules, while the dense structure enhanced ion rejection. Increasing the PVAc concentration to 20 wt.%, (ZGM03), significantly improved pore formation. SEM images revealed a higher number of interconnected pores on both the surface and the cross-section. This structural enhancement led to a notable increase in water flux, as the more open and connected pore network facilitated water transport. However, the Pb(II) rejection rate was slightly lower than that of ZGM2, likely due to the increased permeability allowing some lead ions to pass through. Despite expectations, the flux value of ZGM3 (20 wt.% PVA) was slightly higher than that of ZGM4 (30 wt.% PVA), while its Pb(II) rejection rate was 87%, which was 2% lower than that of ZGM4 when tested with a 50 ppm Pb(II) solution. To better understand this trend, SEM micrographs (Figure 11) were analyzed to assess the pore structure and interconnectivity of the membranes. Increasing the PVAc concentration to 30 wt.% was anticipated to enhance porosity. However, a SEM analysis revealed a reduction in mesoporous structures and the formation of macrovoids, likely due to polymer particle agglomeration, which led to the emergence of large voids after the removal of the organic phase [55]. The presence of these macrovoids negatively impacted pore interconnectivity, reducing surface permeability and leading to a lower water flux compared to ZGM3. Additionally, while the structural voids facilitated Pb(II) accommodation, potentially improving rejection efficiency, the accumulation of Pb(II) ions within these voids may have partially blocked water pathways, further restricting water flux. This suggests that excessive Pb(II) adsorption within the macrovoids could have contributed to the observed permeability reduction, limiting the overall membrane performance.

The balanced performance of ZGM3 (20 wt.% PVAc), in terms of Pb(II) rejection and water flux, can be directly linked to its optimized pore structure, as observed in the SEM micrographs and confirmed by BET/BJH analyses. The SEM images (Figure 4) reveal a well-developed network of uniformly distributed mesopores, with fewer macrovoids compared to ZGM4 (30 wt.% PVAc), which exhibited irregular, oversized pores due to polymer agglomeration. This is consistent with the findings by Wang et al. [31], who demonstrated hierarchical pore formation in geopolymer tube membranes. However, they have used a surfactant to build stable foams. In our study, the geopolymer membranes exhibit uniform pore distribution, likely due to the optimized PVAc concentration (20 wt.%). Unlike the ceramic membranes fabricated by Adam et al. [48], which exhibited significant macrovoid formation due to uncontrolled viscosity of the ceramic suspension.

This morphology translates into a more controlled pore interconnectivity in ZGM3, minimizing defects and facilitating uniform flow pathways. BET analysis further supports this observation, showing a specific surface area of 118.3 m^2^/g with 53% mesopores and 10% micropores (Figure 5c,d). The dominance of mesopores in ZGM3 enhances water permeability by reducing resistance to flow, while the presence of micropores provides abundant active sites for Pb(II) adsorption, contributing to the 87% rejection rate observed in filtration tests (Figure 10). Additionally, the well-connected pore network facilitates faster diffusion of Pb(II) ions, which is consistent with the observed adsorption kinetics, where ZGM3 achieved equilibrium uptake more rapidly than membranes with lower or higher PVAc content. This combination of hierarchical pore distribution and interconnectivity explains why ZGM3 strikes the best balance between flux and rejection performance.

The exact separation mechanism of zeolite-based geopolymer filters is generally acknowledged to involve a combination of size exclusion and ion interactions [3]. The geopolymer matrix exhibits cation exchange capacity, containing exchangeable cations within its framework. Upon contact with the membrane, heavy metal cations displace these exchangeable ions due to their greater binding affinity, thereby driving the ion exchange process [3]. A column test study on sorption capacity revealed that geopolymer beads demonstrated a sorption capacity of 45.32 mg/g, which was comparable to the 45.93 mg/g observed for natural zeolite, indicating that the geopolymer maintained its adsorption efficiency despite structural alterations [3]. Additionally, the cation exchange capacity (CEC) of geopolymer beads was measured at 83.96 meq/100 g, closely matching the 83.65 meq/100 g recorded for natural zeolite, further validating these findings.

**Figure 12:**
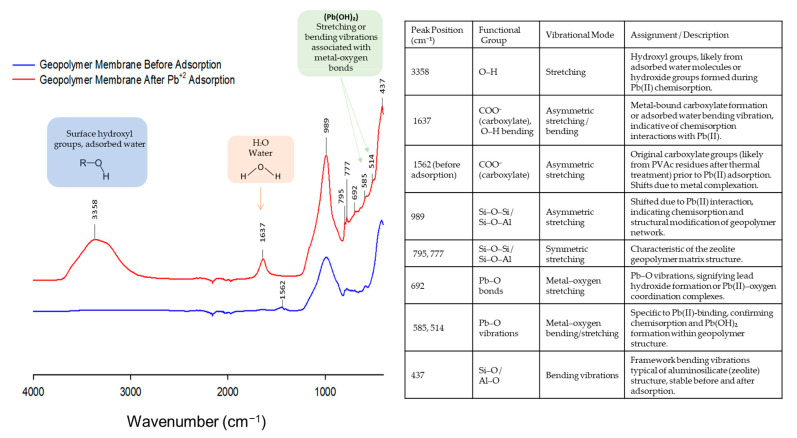
FTIR spectra of zeolite-based geopolymer membranes before and after Pb(II) adsorption, highlighting the chemisorption interactions and associated structural changes. Including a detailed table summary of FTIR peak assignments, including wavenumbers, functional groups, vibrational modes, and descriptions of interactions indicating Pb(II) adsorption onto the geopolymer membrane.

To assess the impact of Pb(II) adsorption on the membrane’s pore structure and surface chemistry, an FTIR analysis was conducted. Figure 12 shows the FTIR spectra of the ZGM before and after Pb(II) adsorption. Following Pb(II) adsorption, a broad and intense band around 3358 cm^−1^ appeared, corresponding to OH-stretching vibrations associated with hydrogen bonding to interlayer water molecules [56]. Additionally, a sharp peak at 1637 cm^−1^ was attributed to bending vibrations of interlayer water molecules, indicating interactions between Pb(II) and the membrane matrix. New shoulders appeared in the FTIR spectra of the geopolymer membrane post-adsorption at around 514 and 692 cm^−1^, suggesting the formation and precipitation of lead hydroxide and the chemisorption mechanism of Pb(II) uptake by the ZGMs [56]. Similar structural changes have been reported for natural zeolites, where bands at 673 and 693 cm^−1^ are associated with ion exchange-induced framework modifications, specifically ring vibrations linked to the SiO_4_ and AlO_4_ tetrahedral structure [52]. Rahmanian et al. [56] also reported comparable spectral changes in layered double hydroxides upon Pb(II) uptake, indicating chemisorption and ion exchange as dominant mechanisms. These findings support the proposed ion exchange process in our ZGM membranes, where Pb(II) displaces other cations within the aluminosilicate framework. These spectral shifts further validate the effective Pb(II) removal mechanism of the zeolite-based geopolymer membrane, which operates through a synergistic combination of ion exchange, surface complexation, and precipitation within the membrane pores.

#### 3.2.2. The Effect of Initial Pb(II) Ions Concentration on the Membrane Performance

**Figure 13:**
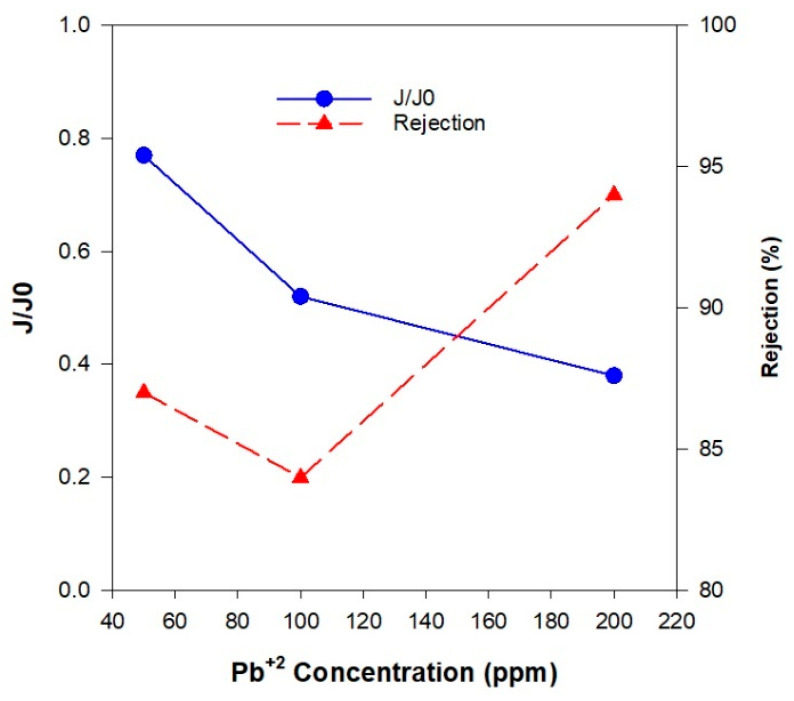
Normalized membrane flux (J/J0, blue circles) and rejection efficiency (%, red triangles) of zeolite-based geopolymer membranes with initial PVAc concentration of 20 wt.%, evaluated at varying Pb(II) concentrations (50, 100, and 200 ppm), at room temperature and neutral pH.

The initial contaminant concentration has a significant impact on membrane performance. Figure 13 presents the normalized flux and rejection efficiency at varying initial Pb(II) concentrations, with the initial PVAc concentration held constant at 20 wt.%. In general, the normalized flux decreased as the Pb(II) ion concentration increased for all membranes, regardless of their porosity. At higher contaminant concentrations, adsorption saturation of the membrane surface occurred more rapidly, resulting in accelerated pore blockage due to the accumulation of Pb(II) ions and other exchanged ions. This blockage restricted the number of available pathways for water permeation, leading to a reduction in flux (Figure 13). Additionally, the increased ionic concentration near the membrane surface led to the formation of a concentrated boundary layer, which may further develop into a gel-like layer on the membrane wall. This phenomenon negatively impacts the permeate flux by establishing a positive concentration gradient toward the membrane surface, thereby increasing the back diffusion of solutes into the bulk solution and reducing net transport across the membrane. The rejection performance varied with the Pb(II) concentration. The highest rejection values for all of the membranes were observed at an initial Pb(II) concentration of 200 ppm, while the lowest rejection occurred at 100 ppm (Table 6).

At an initial Pb(II) concentration of 50 ppm, the ZGM3 membrane exhibited a rejection rate of 87%, which is lower than the 99.9% reported by Zhu et al. [57] for Al_2_O_3_-NaA zeolite composite membranes, which is likely due to the hollow fiber structure and inherently offers a much larger surface area compared to flat-sheet or self-standing membranes. This allows for more contact points between the membrane and the Pb(II)-contaminated water, enhancing adsorption and separation. Additionally, the presence of the NaA zeolite phase, with its smaller pore size (~0.4 nm) and high cation exchange capacity, further enhances Pb(II) removal efficiency [57]. Compared to polymeric membranes such as the PES/GO composites reported by Poolachira and Velmurugan [58], which achieved 80.6% Pb(II) rejection, the zeolite-based geopolymer membranes demonstrate a higher rejection efficiency and a superior mechanical stability. This enhanced performance is likely attributed to the membrane’s combined separation mechanisms of ion exchange and size exclusion. Pb(II) ions are selectively removed by replacing exchangeable cations (Na⁺, K⁺) within the geopolymer framework, while the membrane’s well-defined porous structure restricts ion passage. Furthermore, the negatively charged surface of the geopolymer enhances electrostatic attraction, further improving Pb(II) adsorption efficiency [3].

**Figure 14:**
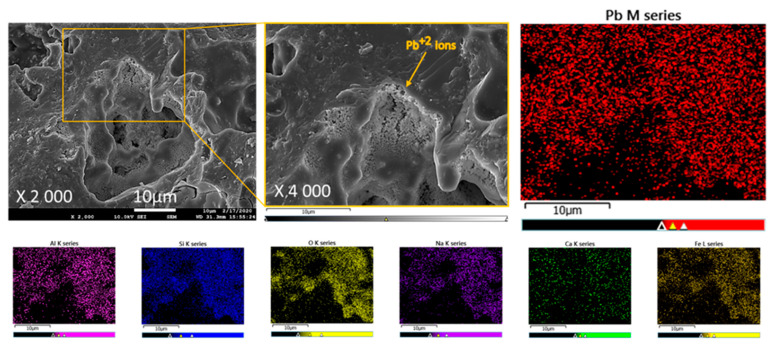
SEM images and corresponding EDS elemental mapping of ZGM prepared with 20 wt.% PVAc after adsorption of Pb(II) at a concentration of 200 ppm.

The flux decreased when the concentration of Pb(II) ions increased from 50 ppm to 100 ppm. However, the rejection efficiency did not improve correspondingly. At this intermediate concentration, partial pore blockage occurred due to the higher number of adsorbed Pb(II) ions, but the reduction in flow rate was not sufficient to prevent the passage of some contaminant ions. This allowed more Pb(II) ions to permeate through the membrane, resulting in lower rejection efficiency despite the slight decrease in flux. In contrast, when the Pb(II) concentration increased further from 100 ppm to 200 ppm, there was a more pronounced reduction in flux, indicating major pore blockage and a significant accumulation of Pb(II) ions on the membrane surface. At this higher concentration, the concentration gradient between the feed and permeate sides became much steeper, providing a greater driving force that facilitated the rapid mass transfer of Pb(II) ions toward the membrane surface. This led to the formation of a dense concentration polarization layer or even a gel-like layer, which acted as an additional selective barrier, effectively restricting the transport of Pb(II) ions through the membrane. As a result, the rejection efficiency increased significantly between 100 ppm and 200 ppm. The element mapping of a typical ZGM was performed using the SEM/EDS analysis (Figure 14) to locate the Pb(II)-accommodated sites. The SEM/EDS images revealed an interconnected porous structure covered with adsorbed Pb(II) particles, particularly concentrated within the structural pores. This observation confirms both successful Pb(II) adsorption and the potential for pore blockage, which contributes to the observed changes in flux and rejection performance.

#### 3.2.3. Comparison of Zeolite-Based Geopolymer Membranes with Alternative Membrane Materials

The following table evaluates various membranes used for heavy metal removal, comparing them with this study in terms of flux-rejection trade-offs, mechanical properties, and material costs. The evaluation of various membranes used for heavy metal removal reveals key differences in terms of flux-rejection trade-offs, mechanical properties, and material costs. While this study focuses on zeolite-based geopolymer membranes, the comparison with other membrane technologies, such as polymeric and ceramic membranes, highlights distinct strengths and limitations. Direct comparisons between polymeric and inorganic membranes are challenging due to the wide variation in polymeric membrane compositions, separation mechanisms, applications, and costs. For meaningful insights, this discussion emphasizes commonly used polymeric membranes like polyvinylidene fluoride (PVDF) and polyether sulfone (PES), which are well-established in water treatment applications [59].

As can be seen in Table 7, the zeolite–geopolymer membrane provides a balanced approach for heavy metal removal, offering a moderate flux and rejection rate combined with strong mechanical strength and a reasonable lifespan. In comparison, ceramic membranes generally outperform in terms of flux and rejection efficiency, making them highly effective for heavy metal removal but with potential trade-offs in cost. Zeolite membranes, known for their excellent removal efficiency and good mechanical strength, are typically more expensive, especially synthetic zeolites, which can make them less economically viable for certain applications. Polymeric membranes, while cost-effective and long-lasting, tend to have lower performance in rejecting heavy metals compared to inorganic membranes. Polymeric membranes, although widely used and cost-effective, tend to underperform in heavy metal rejection and are generally less durable in complex industrial wastewater environments compared to their inorganic counterparts. These membranes are more prone to issues like fouling, scaling, and material degradation, which can undermine their effectiveness over time. Zeolite-based geopolymer membranes are fabricated using inorganic natural resources, such as mined zeolites, resulting in environmentally friendly products. Mined zeolites possess unique properties, like high ion-exchange capacity, porosity, and structural stability, which make them highly promising for various applications, particularly for heavy metal removal within water treatment systems. However, the water treatment application of zeolite heavily depends on the particle size of the natural zeolite. Powdered waste natural zeolite is typically not used in filtration systems due to the high-water pressure drop it causes, which limits its practical application.

## 4. Conclusions

This study developed and investigated novel porous zeolite-based geopolymer membranes for Pb(II) ion adsorption and separation. The membranes were fabricated using waste-grade natural zeolite powder. The membranes initially exhibited limited practical utility due to insufficient porosity and low mechanical strength. To address these issues, a polyvinyl acetate (PVAc) emulsion was employed as a structure-directing agent at varying concentrations and subsequently removed through thermal degradation at 300 °C. This innovative approach resulted in organic-free membranes characterized by enhanced porosity, improved hardness, and mechanical robustness, with annealing effectively healing micro- and macrocracks. Optimal performance in terms of balanced flux and rejection rates was achieved at a 20 wt.% PVAc concentration, while higher concentrations (30 wt.%) introduced macrovoids, compromising membrane integrity. Performance characterization demonstrated that higher Pb(II) ion concentrations led to reduced membrane flux. These geopolymer membranes, leveraging inexpensive raw zeolite without the need for additional support structures, offer cost-effective and sustainable alternatives to conventional inorganic membranes. Despite their advantages, challenges such as membrane scaling and fouling remain, necessitating further optimization of the operational conditions for effective and practical wastewater treatment applications.

Zeolite-based geopolymer membranes were sustainably produced from waste-grade materials, offering significant environmental benefits by reducing waste generation. However, similar to the comparative life cycle assessment (LCA) between zeolite geopolymer and conventional concrete conducted in this study [20], the geopolymer membrane’s sustainability and environmental performance also require quantitative evaluation through LCA. Such an assessment will allow for a detailed comparison between geopolymer membranes and polymeric membranes, addressing environmental impacts across their entire lifecycle, including production, reuse potential, and end-of-life disposal. Evaluating strategies for reuse, recycling, or responsible disposal of these membranes is particularly important, as it ensures alignment with zero-waste objectives and promotes long-term sustainability.

Reproducibility and consistency in membrane performance across batches are significant challenges in inorganic membrane manufacturing, which is essential for industrial applications. Future research should prioritize ensuring reproducibility, optimizing flux-rejection trade-offs, enhancing chemical and mechanical stability, and broadening the application of these membranes as sustainable filtration materials for complex industrial wastewater, especially multi-contaminant systems containing heavy metals and organic pollutants. Additionally, studies focusing on scalability, the optimization of manufacturing processes, and the economic feasibility of zeolite–geopolymer membranes are crucial to facilitate their widespread implementation in industrial water treatment.

## Figures and Tables

**Table 1:** Comparison of various inorganic membranes based on performance, applications, and limitations [10].

Membrane Type	Advantages	Disadvantages	Key Applications
Metal Oxide Ceramic Membranes	High thermal and chemical stability, suitable for harsh environments, tunable pore sizes	High production costs, complex multi-step fabrication, high-temperature sintering, reproducibility challenges	Wastewater treatment, gas separation, pervaporation, nanofiltration
Zeolite Membranes	Molecular sieving properties, high salt rejection, chemical stability, eliminates costly pretreatment of polymeric RO membranes, tunable hydrophilicity, potential natural source	Fragile support layers, poor compatibility between the zeolite and substrate, difficult large-scale fabrication, presence of intercrystalline pores, microdefects	Water purification, desalination, gas separation, pervaporation
Metal Organic Framework (MOF) Membranes	High surface area, tunable porosity, low-temperature synthesis, potential for polymer support, mechanically robust, lower temperature activation than zeolites	High production costs, Limited industrial application, high synthesis complexity, may suffer from structural instability in aqueous environments	Gas separation, water purification, solvent recovery, catalysis
Carbon Nanotube (CNT) Membranes	High surface area, antimicrobial activity, ultra-fast water transport, antimicrobial properties, tunable pore size, compelling mechanical strength	Difficult large-scale production, high cost of synthesis, reproducibility issues	Desalination, wastewater treatment, antimicrobial filtration, molecular separation
Graphene-Based Membranes	High water flux, ultra-thin structure, strong chemical resistance, potential for molecular sieving	Challenges in creating uniform nanopores, reproducibility, limited large-scale availability	Desalination, reverse osmosis, molecular sieving, gas separation

**Table 2:** Geopolymer materials used for the adsorption of heavy metal cations [35].

Source Material	Contaminant	Removal (mg/g)	Adsorbent Dosage/L	Adsorption Conditions
Rice husk ash-based geopolymer	Cu(II)	126.26	0.1 g	pH 8, 25 °C
Metakaolin-based geopolymer	Cu(II)	40.9	0.1 g	pH 8, 25 °C
Blast furnace slag-based geopolymer	As(III)	0.52	25 g	pH 6, 20–23 °C
Metakaolin-based geopolymer	Mn(II)	72.34	0.08 g	pH 6, 30 °C
Rice husk ash-based porous geopolymer	Hg(II)	232.60	0.08 g	pH 6, 25 °C
Lateritic clay-based geopolymer	Co(II)	500	0.5 g	pH 7, 60 °C
Metakaolin-based geopolymer	Pb(II)	86.2	0.1 g	pH 5, 45 °C
Rice husk ash-based porous geopolymer	Pb(II)	312.5	0.08 g	pH 6, 25 °C
Blast furnace slag-based geopolymer	Pb(II)	137.49	2 g	pH 6.5, 25 °C
Natural zeolite-based geopolymer [3]	Pb(II)	45.32	2 g	pH 7.1, 25 °C

**Table 4:** Membrane codes and variables examined in preparing geopolymer samples.

Membrane Code	Sodium Hydroxide Solution (40% *w*/*w*)	Sodium Silicate (55.4–56.4% *w*/*w*)	Natural Zeolite Powder	PVAc Emulsion Concentration	PVAc Emulsion Volume
ZGM1	40 g	50 g	100 g	10 wt.%	10 mL
ZGM2	40 g	50 g	100 g	20 wt.%	10 mL
ZGM3	40 g	50 g	100 g	30 wt.%	10 mL

**Table 5:** Identified crystalline phases, PDF #, composition, and relative abundance in Zeolite and geopolymer samples.

Phase	PDF #	Formula	SiO_2_/Al_2_O_3_ Ratio	Zeolite (wt.%)	Geopolymer (wt.%)
Quartz	98-000-0369	SiO_2_	--	20	10.3
Plagioclase					
-Albite	01-075-1142	NaAlSi_3_O_8_	3.4	3.5	5.3
-Andesine	05-001-0801	Na_0.51_Ca_0.49_(Si_2.56_Al_1.44_O_8_)	2		
-Anorthite	00-012-0301	CaAl_2_Si_2_O_8_	1.13		
K–Feldspar					
-Orthoclase	00-019-0931	KAlSi_3_O_8_	3.4	5.5	2.1
-Sanidine	00-010-0353	KAlSi_3_O_8_	3.4		
Chabazite	01-088-1263	Ca_1.76_Al_3.60_Si_8.40_O_24_·(H_2_O)_9.87_	2.6	0.2	0.3
Clinoptilolite–Heulandite	00-039-1383	KNa_2_Ca_2_(Si_29_Al_7_)O_72_·24H_2_O	4.1	28.1	7.2
Mordenite	00-029-1257	(Ca,Na_2_,K_2_)Al_2_Si_10_O_24_·7H_2_O	5.7	2.2	1
Stilbite–Na	00-039-0223	NaCa_4_(Si_27_Al_9_)O_72_·28(H_2_O)	4.6	19.1	1
Unidentified Amorphous	--	--	--	21.4	72.8

**Table 6:** Flux performance and ion rejection efficiency of zeolite-based geopolymer membranes (ZGMs) evaluated at varying Pb(II) concentrations (50, 100, and 200 ppm).

**Membrane Code**	**ZGM2**	**ZGM3**	**ZGM4**
Pb(II) concentration (ppm)	Flux(L/m^2^.h)		
50	61.9	78.5	41.2
100	38.6	42.5	40.7
200	33.7	35.8	35.1
Pb(II) concentration (ppm)	Rejection (%)		
50	91	87	89
100	88	84	86
200	97	94	96

**Table 7:** High-level comparison of inorganic membranes characterization.

Membrane Type	Contaminant	Flux (L/m^2^·h)	Rejection Rate (%)	Mechanical Strength (MPa)	Industrial-Scale Cost (USD/m^2^)	Life Span (Years)	Mean Pore Size
**Polymeric Membranes**							
PVDF (/PAN *) [60]	Pb(II)	186.14	52.11	-	10–40	1.8–5 [61]	0.1–0.5 µm
Poly ether sulfone [36,58]		18.69	38.9	-	50–65	2–5 [62]	0.1–5 µm [63]
				
**Ceramic Membranes**							
Metal oxide Membranes							
Kaolin–Dolomite [64]	Pb(II)	246.78–1738.56	99.12	1–17 MPa			15–19 nm
Zeolite Membranes							
Al_2_O_3_-NaA zeolite [57]	Pb(II)	727.5	99.9	85.8 ± 3.1	40–66 [65]	5–8	0.41 nm
Metal–Organic Framework Membranes							
ZIF-300 MOF membrane [64]	Cu(II)	39.2	99.21	-	50–200	-	7.9 Å
Zeolite–Geopolymer Membrane (This Study)	Pb(II)	78.5	87%	~180	~20.6	5–8	2–5 nm & >5 nm
**Carbon-Based Membranes**							
Graphene					-	-	
(PSF + GO + DMF) [65]	Pb(II)	100	>90	20			30–400 Å
Carbon Nanotubes [2]	Pb(II)	-	99.99	50	-	-	18.8–23.7 μm
**Hybrid Membranes**							
PES/PVP **/GO *** [58]	Pb(II)	150.21	80.6	-	50–65	-	-

* Polyacrylonitrile. ** Polyvinylpyrrolidone. *** Graphene oxide.

## Data Availability

All data are available within the manuscript.

## Abbreviations

The following abbreviations are used in this manuscript:

A	Membrane surface area
BET	Brunauer–Emmett–Teller
BJH	Barrett–Joyner–Halenda
CARF	Central Analytical Research Facility
DSC	Differential Scanning Calorimetry
EDS	Energy-Dispersive Spectroscopy
FTIR	Fourier-Transform Infrared Spectroscopy
ICP-OES	Inductively Coupled Plasma Optical Emission Spectroscopy
IUPAC	International Union of Pure and Applied Chemistry
J	Water Flux
MDPI	Multidisciplinary Digital Publishing Institute
MPa	Megapascal
PVAC	Polyvinyl Acetate
QUT	Queensland University of Technology
R	Removal efficiency
SEM	Scanning Electron Microscopy
SiO_2_/Al_2_O_3_	Silicon-to-Aluminum Ratio
TGA	Thermogravimetric Analysis
t	Time
XRD	X-ray Diffraction
ZGM	Zeolite Geopolymer Membrane
